# An Improvement Method for Improving the Surface Defect Detection of Industrial Products Based on Contour Matching Algorithms

**DOI:** 10.3390/s24123932

**Published:** 2024-06-17

**Authors:** Haorong Wu, Ziqi Luo, Fuchun Sun, Xiaoxiao Li, Yongxin Zhao

**Affiliations:** 1 School of Electronic Information and Electrical Engineering, Chengdu University, Chengdu 610106, China; luoziqi@stu.cdu.edu.cn (Z.L.); zyx-she@163.com (Y.Z.); 2 School of Mechanical Engineering, Chengdu University, Chengdu 610106, China; sfc@cdu.edu.cn; 3 Key Laboratory of Pattern Recognition and Intelligent Information Processing, Institutions of Higher Education of Sichuan Province, Chengdu University, Chengdu 610106, China; lixiaoxiao@cdu.edu.cn

**Keywords:** contour matching, surface defect detection, pyramid optimization, image sensor

## Abstract

Aiming at the problems of the poor robustness and universality of traditional contour matching algorithms in engineering applications, a method for improving the surface defect detection of industrial products based on contour matching algorithms is detailed in this paper. Based on the image pyramid optimization method, a three-level matching method is designed, which can quickly obtain the candidate pose of the target contour at the top of the image pyramid, combining the integral graph and the integration graph acceleration strategy based on weak classification. It can quickly obtain the rough positioning and rough angle of the target contour, which greatly improves the performance of the algorithm. In addition, to solve the problem that a large number of duplicate candidate points will be generated when the target candidate points are expanded, a method to obtain the optimal candidate points in the neighborhood of the target candidate points is designed, which can guarantee the matching accuracy and greatly reduce the calculation amount. In order to verify the effectiveness of the algorithm, functional test experiments were designed for template building function and contour matching function, including uniform illumination condition, nonlinear condition and contour matching detection under different conditions. The results show that: (1) Under uniform illumination conditions, the detection accuracy can be maintained at about 93%. (2) Under nonlinear illumination conditions, the detection accuracy can be maintained at about 91.84%. (3) When there is an external interference source, there will be a false detection or no detection, and the overall defect detection rate remains above 94%. It is verified that the proposed method can meet the application requirements of common defect detection, and has good robustness and meets the expected functional requirements of the algorithm, providing a strong technical guarantee and data support for the design of embedded image sensors in the later stage.

## 1. Introduction

In view of the advantages of low cost, high reliability, high efficiency and the flexible use of machine vision inspection, it has been widely used in industrial product inspection, involved in processes such as mechanical part defect detection, printing quality detection, and workpiece shape similarity detection [1]. In recent years, more and more researchers have proposed automatic pipeline defect detection algorithms based on machine vision. The traditional image processing algorithms used have relatively low requirements on computing resources, and each process is relatively controllable, able to carry out targeted processing in different environments and making the real-time online detection of product defects under industrial environmental conditions a reality [2]. Using machine vision to realize product defect detection is based on image matching technology (also known as template matching technology) in traditional digital image processing. At present, the template matching algorithm based on the gray value, the image matching algorithm based on feature point, the image moment feature matching algorithm and object detection technology based on deep learning are widely used and researched [3].

The template matching algorithm based on the gray value uses the gray value of a gray image as the feature to match, and has high matching accuracy. However, all the pixels of the entire image need to be calculated as features, which is a huge amount of calculation. At present, the most used template matching algorithms based on the gray value are the MAD and NCC algorithms. The MAD algorithm selected the average sum of the absolute values of the difference between the gray pixel values of the neutron map and the template image as the feature. The algorithm is simple and easy to implement, and the matching accuracy is very high, but the computation is too large. Because of the simple features of this method, the algorithm is very sensitive to noise, especially to light intensity, and is not suitable for use in industrial environment [4]. In order to solve the problem that gray scale template matching algorithm is sensitive to noise, light brightness and image geometric distortion, J. P. Lewis proposed an NCC algorithm [5]. The NCC algorithm selects the similarity between the template image and the image to be matched as the similarity degree value, calculating the correlation coefficient between the subgraph of the image to be matched and the template image in each sliding window range, and finally obtains the correlation coefficient matrix of the subgraph and the template image. When the NCC is closer to 1, it indicates that the correlation between the two images is greater, that is, the similarity between the two images is higher. However, this method still has the problems of high time complexity and high requirement of image size consistency.

The feature points-based contour matching algorithm (SIFT algorithm) was proposed by David Lowe in 1999, which takes the contour with rotation invariance, scaling invariance and luminance invariance as the template feature. Even in complex environments, contour template matching using SIFT features can also achieve a good matching performance [6]. The reason why SIFT has these advantages is that SIFT features are extracted from relatively obvious and prominent areas in the image, and these features are not affected by lighting, affine transformation, noise, and other factors, and it also has a good matching effect when the target is partially blocked or the background is complex. However, the SIFT algorithm has higher requirements for template images, such as the need for a relatively rich texture in the image to construct a sufficiently distinguishable feature vector [7]. If the texture is insufficient, the feature vector distinction is not high, and it is easy to cause false detection. In production, if the contours to be matched cannot be guaranteed to have enough texture features, the SIFT algorithm will not have good universality.

The contour matching algorithm based on the Hu moment was proposed by Hu MK in 1962. It is a method to describe image contour features by using a set of linear combination parameters with normalized center distance. With invariant scaling, rotation and image mapping, it can be used for image recognition and template matching operations [8]. The Hu moment consists of seven normalized central moments, but the contour matching algorithm based on the Hu moment only uses the third order moment. This method cannot describe the image accurately, resulting in the algorithm precision being too low, and the local information of the image cannot be captured. Therefore, as a rough matching algorithm, it is generally suitable for application scenarios with a single contour shape or simple texture features, while it is not suitable for complex scenarios, such as outdoor environment or industrial detection environment [9].

Object detection based on deep learning is a new technology in recent years. Object detection is closely related to contour matching [10]. Object detection involves obtaining the existence, classification and pose status of the object to be detected in an image, while contour matching is used to further judge the similarity between the object and the template on this basis. Object detection in deep learning can use end-to-end thinking to directly locate the target, and train the neural network through a large amount of data and target box labels, so that the neural network can learn to distinguish the background area and the target area in the picture. Then, the target region is selected as the candidate region, and finally the candidate region is extracted for feature detection [11]. At present, the neural networks commonly used in target detection are R-CNN, Fast R-CNN and YOLO [12,13]. Deep learning can quickly realize the target detection of new images with good detection effect, and has the following characteristics [14]: (1) It requires a large number of sample data to train the network (the deeper the level of the network structure, the more data sets need to be trained), but it is difficult to obtain enough defect data sets. (2) High hardware requirements. Deep neural networks require high-end GPUs to train data and have high requirements on memory. (3) Detection accuracy mainly depends on the number of sample data and the number of layers of the neural network structure. Generally, the larger the amount of sample data and the more layers of the network structure, the higher the detection accuracy. However, the detection results given by deep learning are not necessarily the most accurate.

In summary, the template matching algorithm based on the gray value has the advantages of simple principles and high precision. However, it is sensitive to noise and has a high time complexity, so it is not suitable for use in situations with real-time requirements. The contour matching algorithm based on the Hu moment and the contour matching algorithm based on feature points have the advantages of rotation invariance, scaling invariance and displacement invariance, and can meet the real-time requirements when the image is well matched. However, the feature of the matching method based on the Hu moment is too simple, and it cannot describe the local feature of the contour accurately. The contour matching algorithm based on feature points needs rich texture information in the image to extract the feature information with sufficient expressiveness. The object detection algorithm based on deep learning has high hardware requirements, large sample data requirements, and weak adaptability to industrial production environment.

After analyzing the characteristics of various template matching algorithms, an improved method of surface defect detection based on the contour matching algorithm is detailed in this paper. The algorithm is based on the image pyramid optimization method, combined with the integration graph and the integration graph acceleration strategy based on weak classification. The candidate pose of the target contour can be quickly obtained in the image pyramid, and the rough positioning and rough angle information of the target contour can be obtained, which greatly improves the performance of the algorithm. In addition, a method to obtain the optimal candidate points in the neighborhood of the target candidate points is designed to solve the problem of the repeated extension of candidate points in the multi-layer matching process of image pyramid. The candidate points can be extended effectively and comprehensively in the pyramidal candidate points set, which can better solve the problem of the repeated calculation of the extended candidate points, and greatly reduce the calculation amount while ensuring the matching accuracy. The proposed contour matching algorithm has the following advantages: (1) The gradient shape is used as the feature, which has no special requirements on the image, and has the advantages of high precision, good illumination robustness, and occlusion robustness. (2) It has an accurate similarity measure, which can give the similarity score of the matching result and the target. (3) Although the gradient shape feature does not have advanced characteristics, such as rotation invariance, the contour rotation angle can be obtained by traversing all rotation angles, and reasonable and effective optimization of the algorithm can meet the real-time requirements.

## 2. Design of Contour Template Building Algorithm

**Figure 1:**
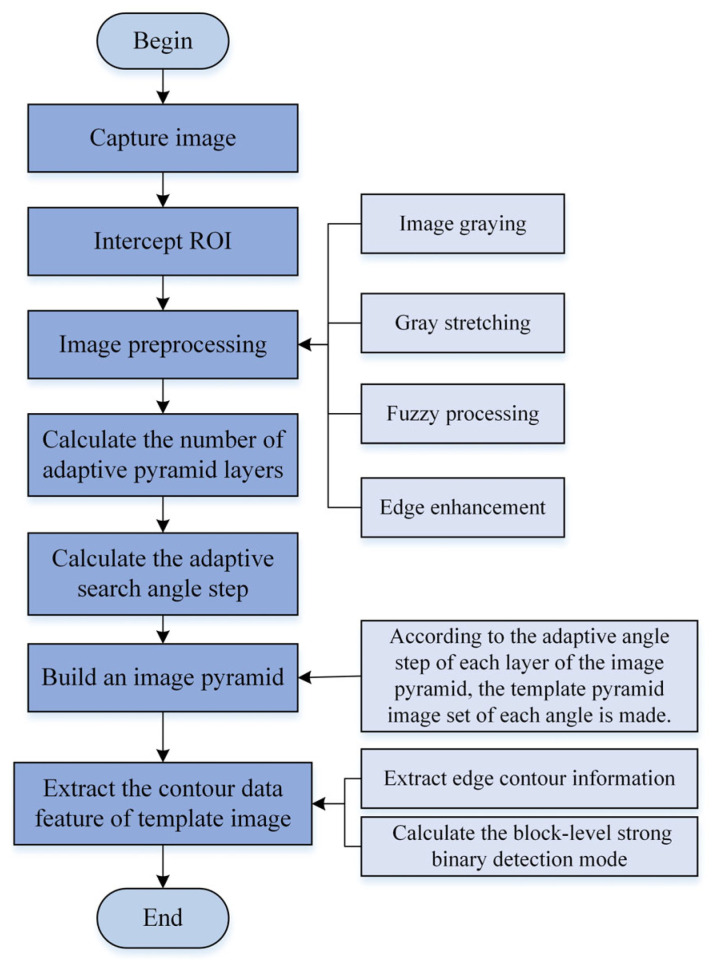
Flowchart of establishing the contour template.

The effect of contour matching is closely related to the quality of the contour template [15]. This section describes the algorithm flow and the key points of contour template creation, including image preprocessing, calculating the number of adaptive pyramid layers, calculating the adaptive search angle step of the image pyramid, establishing the template image pyramid, and extracting the contour feature information of the template image. The process of creating the contour template is shown in Figure 1.

### 2.1. Image Preprocessing

Image preprocessing is a very important step in visual inspection, which is mainly used to improve the image quality, and the image quality has a great impact on the recognition effect of the algorithm. The preprocessing methods used in the contour template building stage include grayscale, grayscale stretching, blur processing and edge sharpening, etc. The specific process of preprocessing is as follows:

(1) Grayscale processing: the three-channel color image is converted into a gray single-channel image, that is, the three-channel color image composed of R, G and B components is converted into a single-channel gray value image composed within the 0~255 range.
(2) Grayscale stretching processing: grayscale stretching can improve the darker areas of an image or the lighter areas of the image, making the contour boundaries of the image more pronounced.
(3) Gaussian blur processing: Gaussian blur processing eliminates the noise in the image, avoids the edge points formed by noise affecting the establishment of the contour template, and then affects the final matching similarity.
(4) Image sharpening processing: The Laplacian operator is used to sharpen the image. If the pixel value of the target point is lower than the average value of the pixels in its neighborhood, the pixel value of the point should be further reduced. If the pixel gray value of this point is higher than the average gray value of other points in its neighborhood, the gray value of this point needs to be further improved.

### 2.2. Gaussian Image Pyramid and Adaptive Parameter Calculation

#### 2.2.1. Establishing Gaussian Image Pyramid

The Gaussian image pyramid is an image processing technique that generates a series of images with different resolutions by continuously shrinking or enlarging the original image. It can effectively handle scale changes in images, reduce noise, extract features, and has high computational efficiency, making it widely used in the fields of image processing and computer vision [16]. Assuming that the height and width of the image to be matched are *w* and *h*, the number of contour points is *K*, and the rotation angle range is *θ*, the time complexity required to traverse the entire image for the contour similarity calculation is *O* = *K* × *θ* × *w* × *h*. The solution search space of the visible algorithm is very large, and if the image is searched exhaustively, the time complexity is too high.

When using Gaussian image pyramids for contour similarity calculation, the time calculation complexity corresponding to different layers of image pyramids varies. Usually, the bottom image of a pyramid is the original image, while the top image is the image with the smallest resolution. Assuming the number of pyramid layers is *i*, the height and width of the top image of the pyramid are *w*/2*^i^*^−1^ and *h*/2*^i^*^−1^, respectively, and the number of contour points *K_i_* is much smaller than that of the bottom image of the pyramid. When using the top level image of a pyramid for the contour similarity calculation, the required time complexity is *O_i_* = *K_i_* × *θ_i_* × *w/2^i^*^−1^ × *h/2^i^*^−1^. Its time complexity is much smaller than that of the original image, which can greatly reduce the search space for solutions, accelerate the matching process, and improve the real-time performance of the algorithm.

#### 2.2.2. Adaptive Pyramid Layers

**Figure 2:**
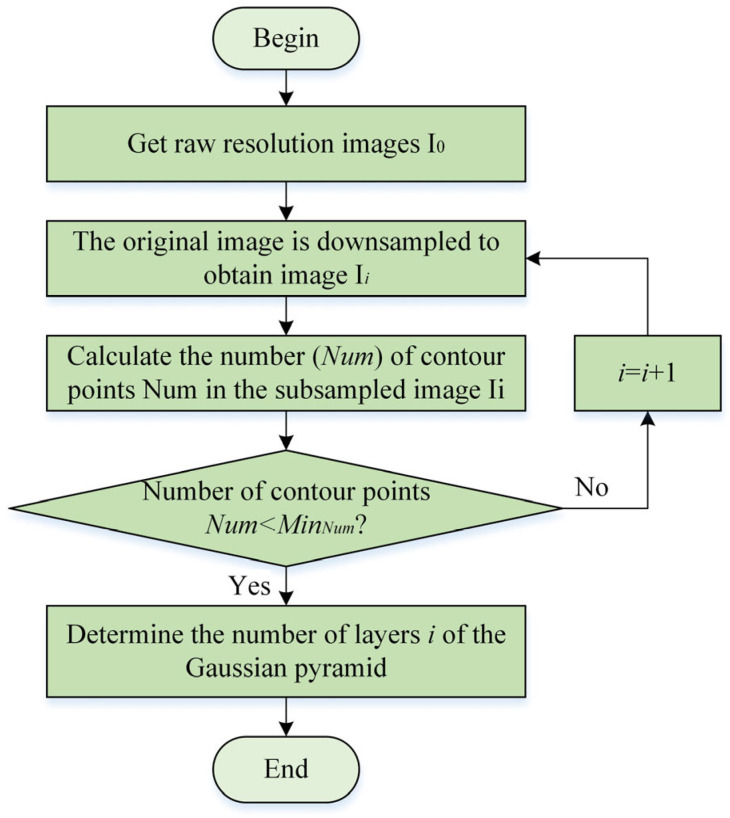
Calculation flowchart of adaptive pyramid layers.

Image pyramids can significantly optimize the time complexity of algorithms, but the choice of pyramid layers has a significant impact on the effectiveness of the algorithm. When constructing an image pyramid, the number of layers should not be uniformly set due to the different characteristics of industrial products [17]. The selection of pyramid layers is actually a trade-off between algorithm availability and algorithm performance. If the number of layers in the pyramid is too low, the image resolution of the top pyramid is still high, and it takes a lot of time to traverse and search for matching. If the number of layers in the pyramid is too high, the loss of contour information on the top layer is severe, resulting in the top layer being unable to match the target position, thereby affecting the matching effect [18]. Figure 2 shows the flowchart for calculating the number of adaptive pyramid layers. This algorithm can determine the number of layers of the image pyramid based on the number of contour points in the top level image of the pyramid, which can ensure the recognition accuracy of the top level pyramid image and improve the usability and efficiency of the algorithm. After multiple experimental analyses, this article found that good results can be achieved when the number of contour points in the top layer image of the pyramid is between 30~50, which can ensure the search speed of the top layer and also retrieve the target position.

### 2.3. Adaptive Angle Step

After generating the image pyramid, it is necessary to rotate the pyramid images of each layer according to a certain angle step size to generate template images for each angle. If the angle step size is too large, it will lead to a small number of established template contours and low matching accuracy. If the angle step size is too small, there are too many templates, and the matching time is too long, it cannot meet the requirements of real-time detection. The paper uses the adaptive angle step calculation method proposed in reference [16], which can calculate the rotation angle step of the contour template based on the distance from the farthest edge point in the template image to the centroid. It is not difficult to find that the resolution of the top image of the pyramid is low, the rotation angle is large, and the number of templates that need to be searched is small, whereas the resolution of the image at the bottom of the pyramid is high, the rotation angle is small, and there are many templates that need to be searched.

### 2.4. Extracting Image Template Features

**Figure 3:**
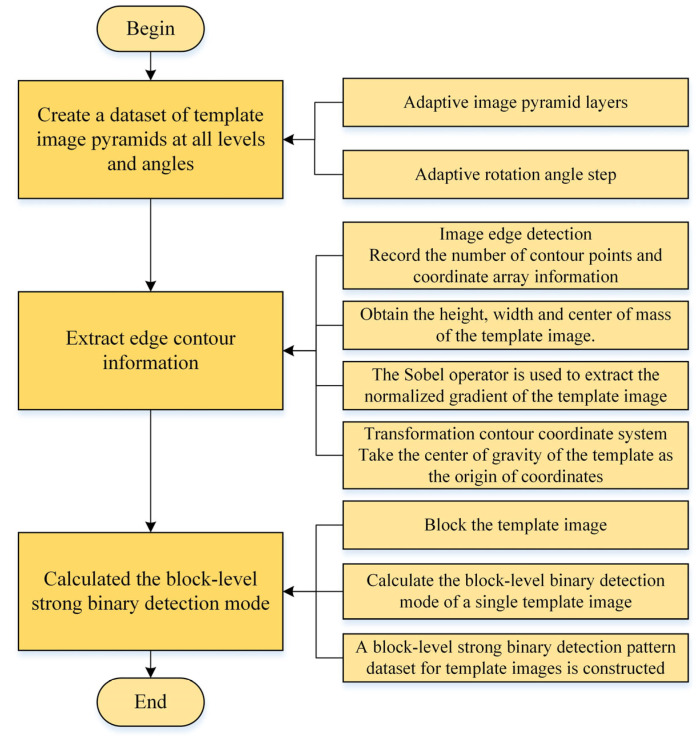
Extraction of template image features sub-process.

The sub-process stage of extracting the template image features is the main time-consuming part of the template establishment stage. At this stage, a large amount of gradient information needs to be calculated, and the gradients need to be normalized and recorded. The sub-process of the calculation is shown in Figure 3.

#### 2.4.1. Extracting Edge Contour Information

The edge contour information of template image includes: the size of the template image, the coordinate point of the edge contour, the center of mass, the normalized gradient vector of edge contour, etc. [16]. First, the template images of each layer and angle template image pyramid data are read one by one. Canny operator is used to extract the edge information of the template image, obtain the coordinates of the interested contour points, and record the height and width of the template image. Then, the Sobel operator is used to obtain the gradient vector of the template image in *x* and *y* directions. It is necessary to traverse the information of all the contour points and calculate the normalized gradient vector information of the corresponding position of the contour points, and the centroid coordinates of the contour shape. Finally, the coordinates of all the edge contour points are transformed by using centroid coordinates, and the point coordinates of each template contour at each angle of each layer are converted to the coordinate system, with centroid as the origin.

#### 2.4.2. Calculating the Block-Level Strong Binary Detection Mode

The time complexity of the traditional contour matching algorithm is too large, so it can not be directly used in the scene with real-time demand. Therefore, Tirui Wu [19] proposed an accelerated method of contour template matching based on weak classification. By using the block-level binary detection mode, based on the integral graph as the feature of the image, this method can quickly skip the non-target background area of the image when the sliding window traverses the image. In this paper, the block-level binary detection mode is also used as the image feature. Firstly, different block sizes are set according to the number of Gaussian pyramid layers, and then the feature of the block-level binary detection mode of each template image in the pyramid template image dataset is quickly calculated using the integral graph. Finally, the block-level binary detection pattern features of all template images are combined to form a block-level strong binary detection pattern dataset, to ensure that the matching process can stop the strategy in advance and quickly skip the low probability of the target.

**Figure 4:**
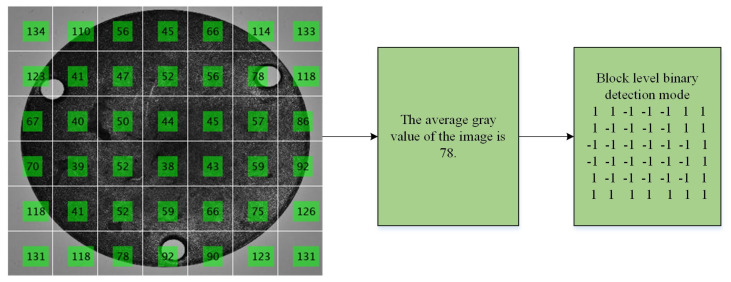
The calculation process of block-level strong binary detection mode.

The value of the block-level binary detection mode feature is calculated from the mean value of the local pixels. The sum of pixel values in a local rectangular area can be quickly obtained by using the integral graph, so the block-level weak binary detection mode eigenvalues can be calculated in constant time. The block-level strong binary detection mode in this paper is composed of a set of block-level weak binary detection modes. Assuming that the size of the template image T is *n* × *n*, the template image T is divided into multiple equal size image blocks of *k* × *l*, and each block corresponds to a block-level binary detection mode. The calculation formula of the block-level binary detection mode is as follows:

(1) $$C_{j}^{T}=\left\{\begin{array}{c} \;1,\;\mu_{i}>\mu \\ -1,\;\mu_{i}>\mu \end{array}\right.$$

where *μ* represents the average gray level of pixels in the contour template image, and *μ_i_* is the average gray level of pixels in the *i* block. $C_{j}^{T}$ represents the weak-order binary detection mode of the *j*-th template image block in the template. All $C_{j}^{T}$ constitute the block-level strong binary detection mode of the template image. Figure 4 is a schematic diagram of a strong binary detection mode at the computing block level.

## 3. Design of Contour Matching Algorithm

**Figure 5:**
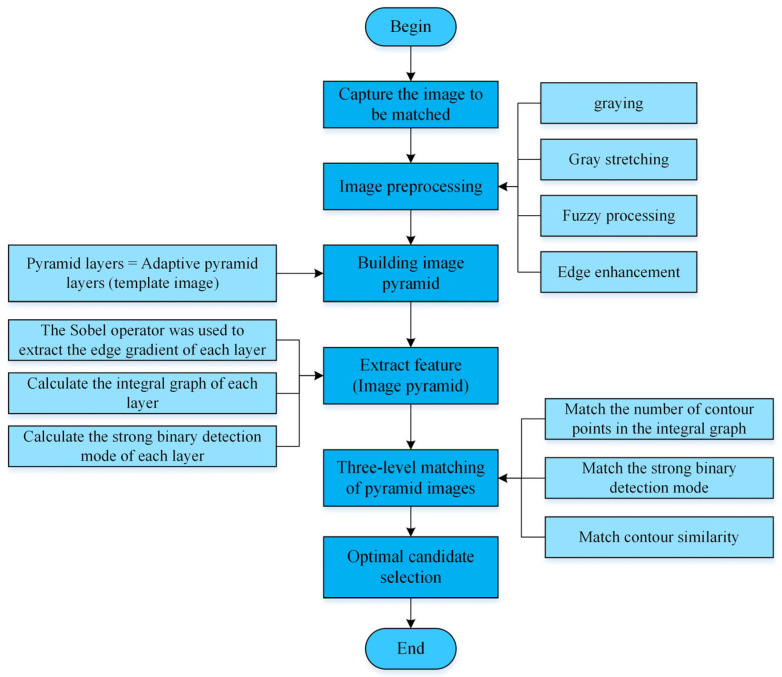
Flowchart of contour matching algorithm.

The contour matching algorithm is the core part of the surface defect detection method of industrial products [20]. This section describes the process based on product contour matching in detail. Figure 5 is the flow chart of the contour matching algorithm. The contour matching algorithm mainly includes six parts: image acquisition, image preprocessing, image pyramid construction, pyramid image feature extraction, pyramid image three-level matching, and best candidate point screening. Among them, the three parts of image preprocessing, image pyramid construction and pyramid image feature extraction are the same as those in the contour template building algorithm in Section 2, and the number of pyramid layers selected is the same as that of the adaptive pyramid layers of the template image, when constructing the gold tower of the template image to be matched.

**Figure 6:**
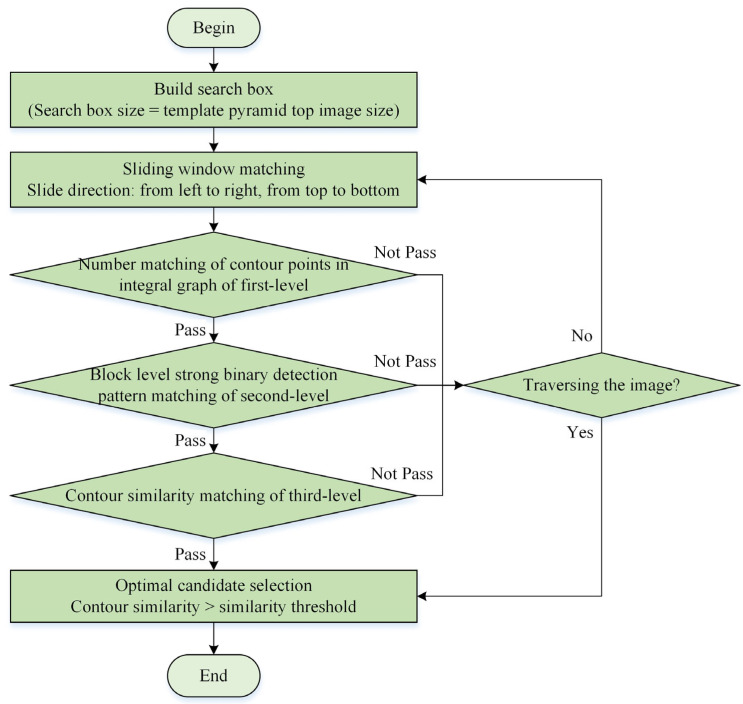
Flowchart of three-level matching process of pyramid image.

The three-level matching method of the pyramid image can be used to locate the contour target quickly. The first-level matching uses the integral graph to quickly calculate the number of all contour points in the target search box, and compares it with the number of contour points in the template on the top of the pyramid. If the difference of contour points is within a certain threshold, the second-level matching stage is entered. Otherwise, go to the next slide window position and start the first-level match again. The two-level matching is realized by using the contour matching acceleration method of the strong binary detection mode. The strong binary detection mode of the image to be matched is compared with the strong binary detection mode of each angle template image. If the number of inconsistent blocks is within a certain threshold, the three-level matching is entered. Otherwise, go to the next slide window position and start again. Three-level matching uses the contour similarity measurement method to calculate the exact similarity. Candidate points can be screened according to the similarity score threshold, and candidate points whose similarity is greater than the threshold can be retained. The specific algorithm flow is shown in Figure 6.

### 3.1. Number Matching of Contour Points in Integral Graph of First-Level

**Figure 7:**
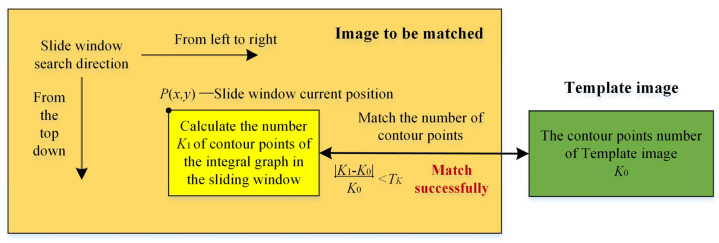
Flowchart of number matching of contour points in integral graph of first-level.

The number matching of contour points in the integral graph of the first-level matching method does not consider the rotation angles, so it is not necessary to match the template images from each angle one by one. The integral graph method is often used to accelerate the template matching algorithm, which can improve the matching efficiency and robustness [20]. Especially when the sliding window traverses the image, it can quickly skip the non-target background area. In this paper, the number of contour points *K*_1_ of the image to be matched with any rectangular sliding window is quickly calculated with the number of contour points *K*_0_ of the template image, and a comparison is made between them. If the difference between the number of contour points |*K*_1_ − *K*_0_|/*K*_0_ is less than the threshold *T_K_* (*T_K_* = 0.5), the first-level matching stage passes, and the second-level matching stage is entered. Otherwise, the first-level matching is considered as failed, and it continues to pass through the next sliding window. Figure 7 shows the matching process of the number points of the contour of the first-level integral graph.

### 3.2. Block Level Strong Binary Detection Pattern Matching of Second-Level

**Figure 8:**
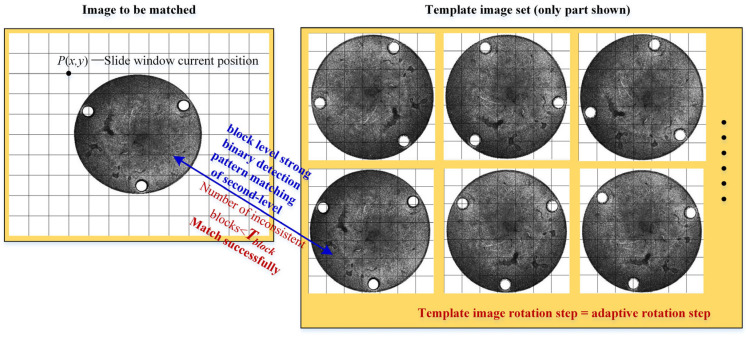
Flowchart of block level strong binary detection pattern matching of second-level.

The second-level matching needs to consider the rotation angle, to match the template image of all rotation angles on the top image. The number of angles to be matched is determined by the rotation angle step. Generally speaking, the higher the resolution, the more angles to be matched. The rotation angle step here is the adaptive search angle step in Section 2.3. The second-level matching uses the integrated graph strong binary detection pattern matching algorithm, but the top pyramid image resolution is generally low, so it is not suitable for accelerating the integrated graph directly on the top pyramid. Different integral block sizes are used to solve this problem. Different block sizes can be set to simulate the image pyramid of different layers by treating the original resolution image of the matching image and the template image. For example, when the block size is 2 × 2, 4 × 4, 8 × 8, 16 × 16, 32 × 32, corresponding to the first layer, the second layer, the third layer, the fourth layer, the fifth layer of the image pyramid, and so on. From Section 2, the number of adaptive layers of the Gaussian image pyramid is known, and the block size of the bottom image of the pyramid can be obtained. Accordingly, the position of pixel coordinates on the original resolution image can be calculated by the corresponding multiple change in pixel coordinates. According to Section 2.4.2, the block-level strong binary detection mode of the image to be matched is obtained, it is matched with the template one by one, and all candidate points meeting the matching conditions are finally found. The process of block-level strong binary detection pattern matching is described as follows, and Figure 8 shows the pattern matching process of two-level block-level strong binary detection.

(1) Obtain the block-level strong binary detection mode of the image to be matched in the sliding window.
(2) Read the block-level strong binary detection mode of each angle template image, and traverse the template block-level strong binary detection mode, corresponding to all rotation angle steps.
(3) The binary detection mode of the sliding window is compared with the binary detection mode of each angle of the template image. If the number of inconsistent blocks is less than the threshold value *T_block_*, the second-level matching passes and the third-level matching stage is entered. Otherwise, compare the binary detection mode of the next angle. It should be noted that Tblock is the maximum allowed number of block inconsistencies. If *T_block_* is selected as a value that is too small, it is sensitive to noise. If the Tblock selection is too large, there will be too many non-candidate points that need to be further matched accurately. Here *T_block_* = total number of blocks in sliding window *0.2.
(4) Complete the comparison of the block-level strong binary detection modes of all angle templates through traversal, and slide the window to the next position.
(5) Traverse the entire image to be matched, compare all candidate points, and select the best candidate points.

### 3.3. Contour Similarity Matching of Third-Level

**Figure 9:**
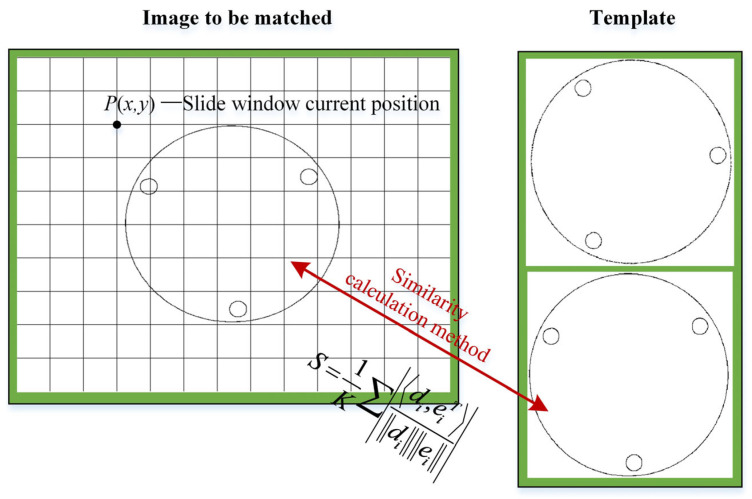
Flowchart of contour similarity matching of third-level.

The main difference between many template matching algorithms is the measurement of image similarity. Carsten Steger [21] proposed a similarity measurement method with good robustness under an occluding, cluttered environment and uneven illumination, which uses the geometric information of images as the main feature. The template consists of a series of edge contour points *p_i_* and the corresponding gradient direction vector *d_i_*, where *p_i_* = (*x_i_*, *y_i_*), *d_i_* = (*t_i_*, *u_i_*), *i* = 1,…, *n*. It should be noted that the edge contour point *p_i_* should be converted to the coordinate system with the centroid of the template contour edge as the origin, and the contour point coordinate *p_i_* and the gradient vector *d_i_* can be extracted by referring to the edge contour information extraction method in Section 2.4.1. The feature of the image to be matched is also composed of the coordinate *p*(*x*, *y*) of the edge contour point and the corresponding gradient vector *e_x,y_* = (*v_x,y_*, *w_x,y_*). Then, the measurement formula of contour similarity can be expressed as:

(2) $$S=\frac{1}{K}\sum \left|\frac{\langle d_{i},e_{i}^{T}\rangle }{\left\|d_{i}\right\|\left\|e_{i}\right\|}\right|$$

where: *K* represents the minimum number of contour points in the image to be matched and the template image. The gradient of the contour points of the template and the corresponding position in the sliding window to be searched is computed by the vector dot product and the average value. Since each vector is normalized, at each corresponding point, if the two directions are exactly the same, the dot product of the vector at that point takes the maximum value 1. If the directions of the two points are completely opposite, the dot product is the minimum value −1. Formula (2) takes the absolute value of the vector dot product result to ensure that the algorithm can match even if the target polarity is reversed. The intensity of the vector is also unitized in the metric formula, which can eliminate the influence caused by the change in light intensity. Figure 9 shows the schematic diagram of three-level matching.

Three-level matching uses the similarity measurement method based on contour edge gradient information to calculate the similarity of candidate points and output it as the result for pyramid non-top-level matching. Therefore, when the three-level matching at the top of the pyramid is completed, it is necessary to output at least 30 candidate points as the candidate point set of the next layer, and expand these candidate points in the neighborhood of angles and positions in the next layer.

### 3.4. Selection of the Best Candidate Points

**Figure 10:**
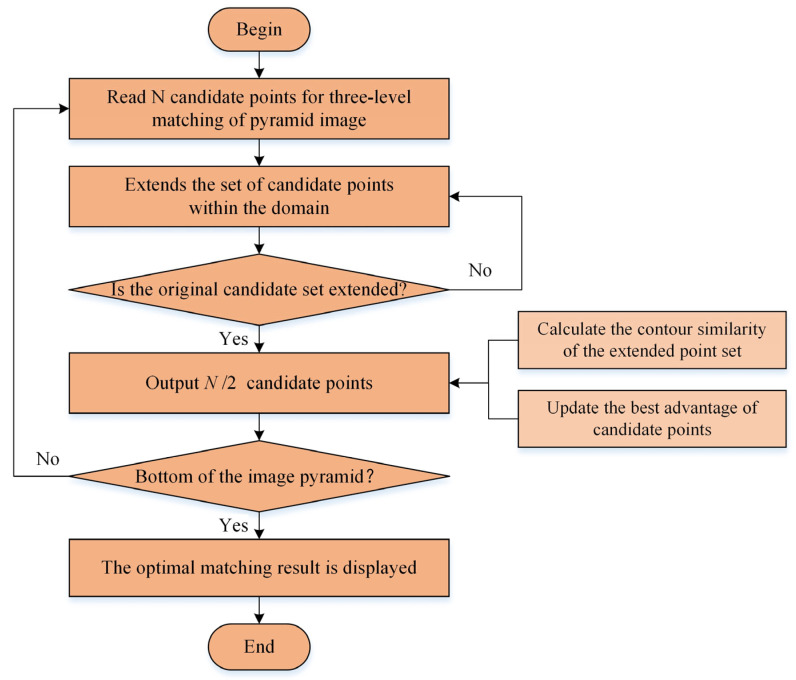
Selection process of the best candidate points.

Since the rough position of candidate points has been obtained on the top layer of the pyramid, it is only necessary to use the similarity method for accurate matching in the non-top layer of the pyramid. Figure 10 shows the flow chart for selecting the best candidate points. In the non-top-level matching process of the pyramid, if *N* candidate points are the output from the previous layer, *ap* angles in the angle neighborhood of each candidate point in the next layer need to be combined with *xp* × *yp* position ranges in the coordinate neighborhood to obtain *ap* × *xp* × *yp* more accurate candidate points. Due to the clustering of candidate points, this *ap* × *xp* × *yp* point will have a large number of duplicate points.

Here, a similar non-maximum suppression method is used to select the optimal candidate points in the neighborhood. First, a matrix of the same size as the image to be matched is used to record the expansion of each candidate point, and the data table of all candidate points is obtained. Then, the candidate points and their extension points are traversed, and the optimal candidate points in the neighborhood of each candidate point are found and used as the new candidate points. Finally, the first *N*/2 candidates with the highest similarity among these optimal new candidates are selected as the input of the next layer of the pyramid.

## 4. Experimental Test and Result Analysis

In this section, functional tests are carried out on the contour template establishment stage and the contour matching stage, respectively. The function test of the contour template building is mainly used to verify whether the information of the edge contour can be correctly extracted under different lighting conditions. The template matching test is carried out to identify good and bad products on the basis of the correct extraction of contour edge information, and to test the feasibility and practicability of the proposed method through matching results.

Two illumination conditions, uniform illumination and nonlinear illumination, were selected for the experiment [22]. Under uniform lighting conditions, due to the uniform distribution of light, the product surface often presents a more ideal image effect. Under nonlinear lighting conditions, due to the change in light, the surface of the product often presents a highlight area and a shadow area. The existence of the highlight area and the shadow area will directly affect the extraction effect of the contour edge. If the edge extraction is not accurate, it will directly affect the matching result, and usually misjudge the good product as a defective product. The parameters in the experiment are shown in Table 1.

### 4.1. Function Test of Template Establishment

Different test variables are tested in the contour template building stage. The template is set up in different environments to test whether the complete characteristic data can be accurately obtained. This mainly tests the extraction effect of the product edge contour, that is, whether the shape edge of the target can be accurately extracted under different conditions.

(1) Uniform lighting conditions

Uniform lighting refers to the lighting effect in which the light is evenly distributed in an area and there is no obvious difference between light and dark. This lighting effect ensures that the entire area is fully illuminated to avoid shadows or uneven light. The function test of the template establishment under uniform illumination conditions is essential to investigate the extraction quality of the template contour under different illumination conditions. Generally, more noise contours are generated under high luminance conditions, while fewer than all of the contour points are often extracted under low luminance conditions. Therefore, comparative experiments under three different lighting conditions were designed, as shown in Table 2. Table 2 shows the contour extraction comparison of the three items under dark, normal and bright light conditions, respectively. It can be found that under normal lighting conditions, the edge information is clear and complete, the noise points are fewer, and all the contour points in the detection box can be extracted very well. Under bright lighting conditions, the edge information is also clear. Due to the influence of light, white noise is easily formed when detecting characters due to reflection, which affects the edge extraction effect. Therefore, bright lighting conditions are suitable for situations with weak edge information and few noise points. Dark lighting conditions are suitable for situations with clear edge information and many noise points.

(2) Nonlinear illumination

The nonlinear illumination is different from the variation in light intensity under uniform illumination conditions, and the illumination on the surface of the product is not uniform. Point light sources can be set separately in the front, back, left, right of the product to simulate nonlinear lighting conditions. Table 3 shows the contour extraction results of the items to be tested under nonlinear lighting conditions. It can be seen that the edge information extracted under the four nonlinear lighting conditions is still clear and complete, with fewer noise points, and all contour points in the detection box can be well extracted.

The template building function test verifies the contour extraction effect of the template image under uniform illumination and nonlinear illumination. Because the experimental conditions are relatively stable, the robustness of the algorithm can be guaranteed during the template building stage, and the ideal edge contour can be extracted under different lighting conditions. However, there are definitely differences between the real production environment and the experimental environment. Therefore, the erasure function of manually configured non-interesting contour points should be added in the subsequent research, so that the contour noise of the template edge formed by the change in illumination can be manually erased to reduce the contour error of the template as much as possible.

### 4.2. Function Test of Contour Matching

The contour matching function test is different from the template establishment function test, which mainly tests whether the image to be matched can be matched with the template image quickly and accurately under various conditions. The contour matching experiment is divided into good product matching experiments and bad product matching experiments (defect detection). The lighting condition is referenced to the template establishment function test experiment, and the detection scenario of complex background (multi-interference source) is added. The product defects mainly include new defects and missing defects, which can be simulated by adding interference sources. The outline matching experimental design scheme is described as follows:

(1) Good product detection under uniform lighting conditions: Under uniform lighting conditions (light intensity is *E* = 50, 100, 150 lux), the images of good products at any angle, any position and with external disturbances are collected, matched with the template, and the qualified products are marked as ‘OK’. It is expected that a good product image to be matched can normally match the template image (such as any template image given in Table 4, Table 5 and Table 6) under uniform lighting conditions.
(2) Good product detection under nonlinear lighting conditions: Under nonlinear lighting conditions, collect any angle, any position and good product image with external interference, match with the template, and mark the qualified product as ‘OK’. It is expected that a good product image to be matched can match the template image (such as any template image given in Table 4, Table 5 and Table 6) normally under nonlinear lighting conditions.
(3) Defect detection: Defect types include new defects, missing defects, external interference occlusion, etc., and the detected unqualified products are marked as “NG” (Not Good). It is expected that the defect can be correctly detected under both uniform illumination and nonlinear illumination.

The products of contour matching experiment include 3-hole stamping parts, mineral water bottle caps, table hole caps, etc. (Only the experimental results of these three products are given here). The experimental conditions and test effects of different test products are shown in Table 4, Table 5 and Table 6. The detection data set of each product contains 1000 images of good products and 500 images of defective products. Defect types include 200 new defects, 200 missing defects, and 100 from external interference (shielding edge), a total of 1500.

**Table 4:** Experimental conditions and detection results of contour matching of 3-hole stamping parts.

Detection Type	Good Product Detection “OK”							Defect Detection “NG”
Experimental Condition	Uniform Illumination Any Angle, Any Position, There Are Distractions			Nonlinear Illumination Any Angle, Any Position, There Are Distractions				Any Illumination Parameters Above
Parameter	E = 50 lux	E = 100 lux	E = 150 lux	Left Illumination	Right Illumination	Front Illumination	Back Illumination	Any Template Image Above
Template image	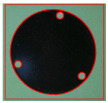	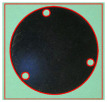	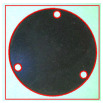	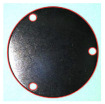	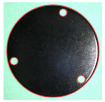	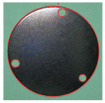	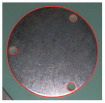	———
Detection results (show part only)	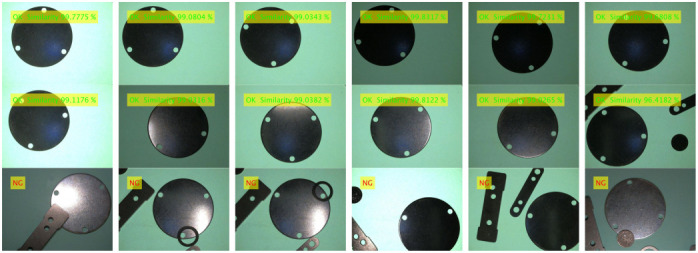							

**Table 5:** Experimental conditions and detection results of contour matching of mineral water bottle caps.

Detection Type	Good Product Detection “OK”							Defect Detection “NG”
Experimental Condition	Uniform Illumination Any Angle, Any Position, There Are Distractions			Nonlinear Illumination Any Angle, Any Position, There Are Distractions				Any Illumination Parameters Above
Parameter	E = 50 lux	E = 100 lux	E = 150 lux	Left Illumination	Right Illumination	Front Illumination	Back Illumination	Any Template Image Above
Template image	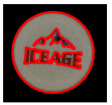	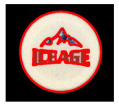	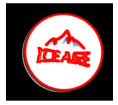	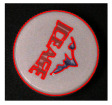	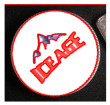	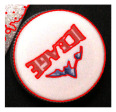	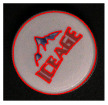	———
Detection results (show part only)	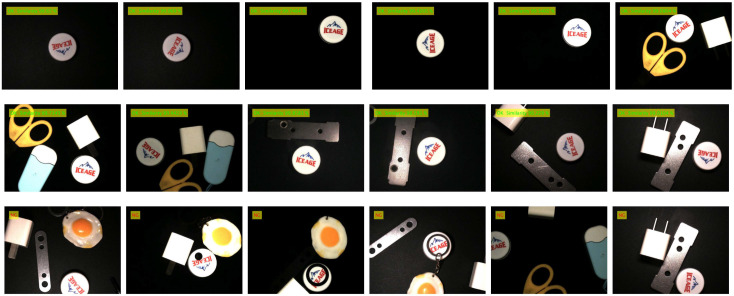							

**Table 6:** Experimental conditions and detection results of contour matching of table hole caps.

Detection Type	Good Product Detection “OK”							Defect Detection “NG”
Experimental Condition	Uniform Illumination Any Angle, Any Position, There Are Distractions			Nonlinear Illumination Any Angle, Any Position, There Are Distractions				Any Illumination Parameters Above
Parameter	E = 50 lux	E = 100 lux	E = 150 lux	Left Illumination	Right Illumination	Front Illumination	Back Illumination	Any Template Image Above
Template image	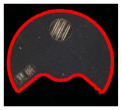	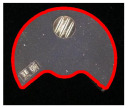	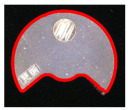	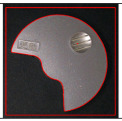	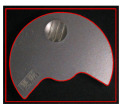	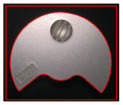	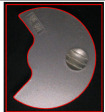	———
Detection results (show part only)	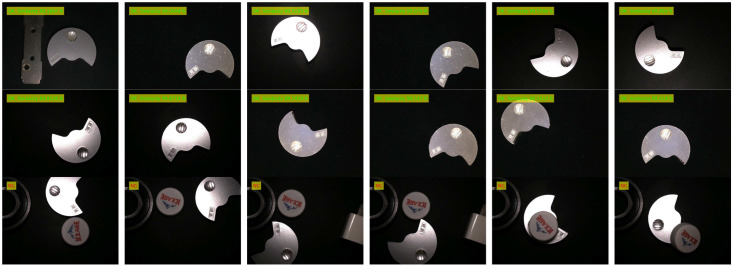							

The contour matching experiments were run on Windows10, AMD Ryzen 536006-Core Processor (3.60 GHz), and MATLAB2022b. The average matching time of the three products under different experimental conditions is shown in Table 7. As can be seen from Table 7, the testing time of the three products is 301.57 ms, 212.00 ms and 284.14 ms, respectively, which can meet the requirements of real-time testing.

In the contour matching experiment, the gray value of the template image and the image to be matched were changed by changing the illumination intensity, so as to verify the normal matching of good products and the accuracy stability of the proposed defect detection method under the condition of the change in the brightness of the template. The accuracy results of the contour matching experiments of 3-hole stamping parts, mineral water bottle caps and table hole caps are shown in Table 8. Here, *Accuracy*, *Precision* and *Recall* are used to evaluate the performance and accuracy of the defect detection algorithm, and Confusion Matrix is used for reference to calculate the above indexes. TP indicates True Positive, TN indicates True Negative, FP indicates False Positive, and FN indicates False Negative. *Accuracy* refers to the proportion of the number of samples correctly classified by the classifier to the total number of samples. *Precision* (good product detection rate) refers to the proportion of samples classified as positive cases that actually negative cases. *Recall* (the defect detection rate) refers to the proportion of samples that are actually positive examples and are correctly classified as positive examples. The calculation formula is as follows:

*Accuracy* = (TP + TN)/(TP + TN + FP + FN) (3)

*Precision* = TP/(TP + FP) (4)

*Recall* = TN/(TN + FN) (5)

**Figure 11:**
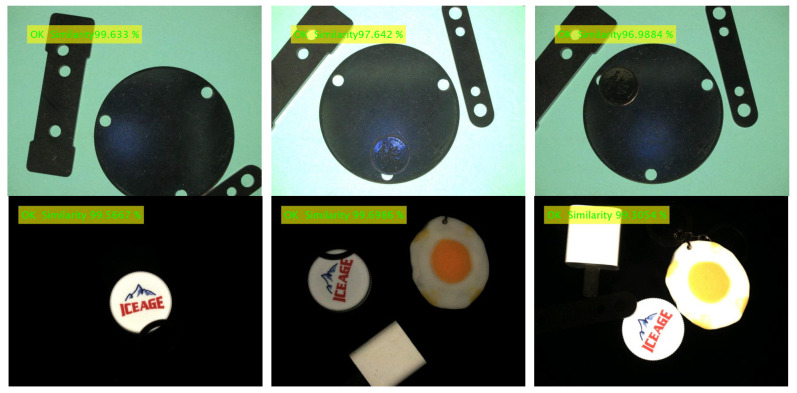
Product false detection caused by interference source occlusion.

From the detection accuracy of the three items in Table 8 for 100 matches under different experimental conditions, it can be seen that:

(1) The average accuracy, precision and recall of the 3-hole metal stamping parts under various experimental conditions were 93.01%, 94.43% and 96.68%, respectively; The average accuracy, precision and recall of the mineral water bottle caps under each experimental condition were 94.44%, 95.07% and 95.62%, respectively. The average accuracy, precision and recall of the table hole cover under each experimental condition were 92.35%, 94.11% and 94.75%, respectively. The average accuracy, good product matching rate and bad product matching rate of product defect detection can be kept above 92%, which proves the effectiveness and practicability of the proposed method.
(2) (2) Under uniform lighting conditions, when the light intensity E = 100 lux, the detection accuracy of the three products is higher than that under dark lighting (E = 50 lux) and bright lighting (E = 150 lux) conditions. The average values of detection accuracy under uniform illumination are 93.60%, 95.74% and 92.68%, respectively, as shown in Table 9. The overall detection accuracy remains basically unchanged, the lowest can be maintained above 92.68%, and a high defect detection rate can be maintained. This is because the contour matching algorithm based on shape features is adopted. The gradient features used are normalized gradient vector features, and the change in light intensity will not directly affect the gradient vector.
(3) Under nonlinear illumination conditions, the image is subjected to nonlinear illumination in different directions, and the gradient direction vector of part of the contour will change compared with the gradient direction of the template. The detection accuracy of the three products fluctuated under nonlinear illumination conditions, and the average detection accuracy was 92.57%, 93.46% and 92.11%, respectively, as shown in Table 9. The overall detection accuracy is basically unchanged, and the defect detection rate is high, which indicates that the nonlinear illumination has little influence on the accuracy of the algorithm.
(4) A statistical analysis was carried out on the comprehensive results of the matching accuracy of three kinds of products matched 100 times. It was found that the standard deviation of detection accuracy, good product matching rate and bad product matching rate were 0.0115, 0.0054 and 0.0106, and the confidence intervals were [92.57, 94.20], [93.56, 94.08] and [94.90, 96.74], respectively. This means that the true average of the experimental results with a standard deviation of 0.0115, 0.0054 and 0.0106 has a 95% probability of falling within the range of [92.57%, 94.20%], [93.56%, 94.58%] and [94.90%, 96.74%], which is consistent with the calculated detection mean value. It shows that the matching results of the proposed method are real and reliable.
(5) In the presence of external interference sources, the outline of the item will be undetected, as shown in Figure 11. Especially when the external interference source is too close to the detected item, there is edge occlusion and there will be mismatching, so try to choose the appropriate template frame size. This paper mainly detects two types of defects: new defects and missing defects. When detecting new defects, if the new defects are not obvious or similar to the gray value of the background, the edge contour and other matching information of the defects cannot be correctly extracted, the algorithm will classify it as the background, and it cannot be correctly detected, as shown in Figure 11. Due to the obvious difference between the contour of the image to be matched and the contour of the template, the algorithm can accurately detect the missing flaw detection. Therefore, the algorithm is more suitable for the detection of missing defects, while the detection rate of new defects is low, and the overall defect detection rate remains at about 96%.

**Table 9:** Mean values of detection accuracy under uniform illumination and nonlinear illumination conditions and confidence interval.

Product	Experimental Condition	Matching Accuracy of 100 Times		
		*Accuracy*	*Precision*	*Recall*
3-hole stamping parts	Uniform illumination	93.60%	94.81%	97.17%
	Nonlinear illumination	92.57%	94.15%	96.32%
	All conditions	93.01%	94.43%	96.69%
Mineral water bottle cap	Uniform illumination	95.74%	95.46%	97.17%
	Nonlinear illumination	93.46%	94.78%	96.05%
	All conditions	94.43%	95.07%	96.53%
Table hole caps	Uniform illumination	92.68%	93.70%	95.70%
	Nonlinear illumination	92.11%	94.42%	94.05%
	All conditions	92.35%	94.11%	94.76%
Statistical analysis results of three kinds of products	Mean value	93.33%	94.55%	96.05%
	Standard deviation	0.0115	0.0054	0.0106
	Confidence interval	[92.57, 94.20]	[93.56, 94.58]	[94.90, 96.74]

## 5. Conclusions

Aiming at the problems of the poor robustness and universality of traditional contour matching algorithms in engineering applications, this paper designs an improved method of surface defect detection for industrial products based on a contour matching algorithm. This method adopts the optimization method based on an image pyramid, combines the three-level matching and the pyramid layer matching optimization, and has a good performance improvement. The implementation of the algorithm mainly includes contour template building and contour template matching, and the three-level matching of pyramid layer and optimization methods, such as the ordering of candidate points in the neighborhood and de-duplication. Finally, in order to test the performance of the algorithm in this paper, the template building and contour matching function test experiments are designed, respectively. The detection accuracy and defect detection rate of good products can reach 93% and 94%, and it has good robustness to uniform illumination and nonlinear illumination conditions.

The contour matching algorithm detailed in this paper can be applied to the defect detection of most industrial products with simple requirements. With the refinement of industrial production requirements and the complexity of the production environment, the algorithm needs to be extended as follows in the later stage: (1) The parameter setting needs to be optimized, the parameter configuration interface will be provided in the later stage, and parameters can be set adaptively and manually according to the application scenario. (2) For the precision measurement requirements of industrial products, high-resolution images need to be targeted for optimization. (3) Combine the deep learning method with this algorithm to improve the accuracy and efficiency of real-time target tracking and detection.

## Figures and Tables

**Table 1:** Functional test scenarios and parameter Settings.

Test Stage	Scenarios	Variables	Parameter	Expectation
Template Establishment	1	Uniform light intensity	E = 50 lux	The same template can be matched normally under different light intensities.
			E = 100 lux	
			E = 150 lux	
	2	Nonlinear illumination	Set a point light source to the left of the product	The same template can be matched normally under different lighting conditions.
			Set a point light source to the right of the product	
			Set a point light source to the front of the product	
			Set a point light source to the back of the product	
Contour Matching	3	Product defect	Added types of defects	Compared with the template, the product to be matched has obvious defects and can be identified normally.
			Missing type of defect	
	4	External interference (Complex background)	Multiple interference sources	Under complex background or external interference conditions, good and defective products can be identified normally.

**Table 2:** Contour extraction test of the product under different light intensities.

Product	Dark Lighting Conditions	Normal Lighting Conditions	Bright Lighting Conditions
3-hole stamping parts	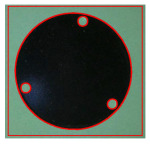	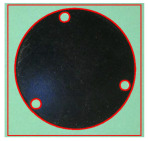	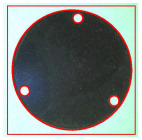
Mineral water bottle cap	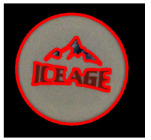	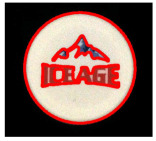	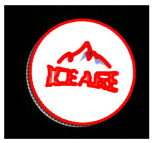
Table hole cover	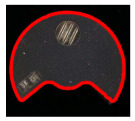	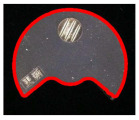	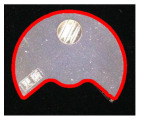

**Table 3:** Contour extraction test of the product under nonlinear illumination conditions.

Product	Left Illumination	Right Illumination	Front Illumination	Back Illumination
3-hole stamping parts	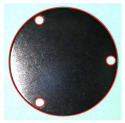	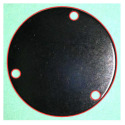	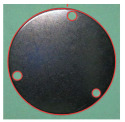	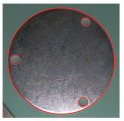
Mineral water bottle cap	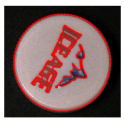	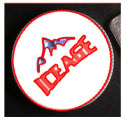	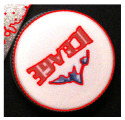	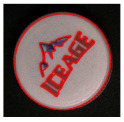
Table hole cover	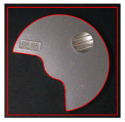	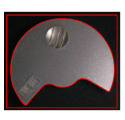	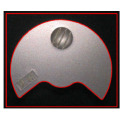	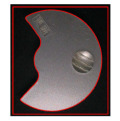

**Table 7:** Average matching time of the three products under different experimental conditions (ms).

	Experimental Condition	Uniform Illumination			Nonlinear Illumination				Mean Time
Product		50 lux	100 lux	150 lux	Left	Right	Front	Back	
3-hole stamping parts		298	290	303	308	306	301	305	301.57
Mineral water bottle cap		213	206	210	209	216	221	209	212.00
Table hole cover		282	262	288	279	291	285	281	284.14

**Table 8:** Detection accuracy of contour matching experiment (100 times matching accuracy).

Product	Experimental Condition	Illumination Parameter	Matching Accuracy of 100 Times		
			*Accuracy*	*Precision*	*Recall*
3-hole stamping parts	Uniform illumination	E = 50 lux	93.21%	94.62%	97.21%
		E = 100 lux	95.59%	96.03%	96.99%
		E = 150 lux	92.01%	93.77%	97.32%
	Nonlinear illumination	Left illumination	91.89%	93.42%	95.88%
		Right illumination	92.36%	94.96%	96.35%
		Front illumination	92.56%	93.85%	95.42%
		Back illumination	93.45%	94.37%	97.63%
Mineral water bottle caps	Uniform illumination	E = 50 lux	95.88%	96.04%	97.21%
		E = 100 lux	97.32%	97.63%	96.99%
		E = 150 lux	94.01%	92.72%	97.32%
	Nonlinear illumination	Left illumination	93.95%	94.78%	97.35%
		Right illumination	92.35%	95.96%	96.91%
		Front illumination	92.87%	94.75%	94.18%
		Back illumination	94.66%	93.61%	95.74%
Table hole caps	Uniform illumination	E = 50 lux	93.45%	94.11%	95.41%
		E = 100 lux	94.37%	95.12%	97.85%
		E = 150 lux	90.21%	91.87%	93.84%
	Nonlinear illumination	Left illumination	92.33%	94.55%	94.63%
		Right illumination	90.68%	93.65%	94.18%
		Front illumination	91.85%	94.69%	93.55%
		Back illumination	93.57%	94.78%	93.84%
Average accuracy of 3-hole stamping parts/Mineral water bottle caps/Table hole caps			93.01%/94.44%/92.35%	94.43%/95.07%/ 94.11%	96.68%/95.62%/94.75%

## Data Availability

The original contributions presented in the study are included in the article. Further inquiries can be directed to the corresponding author.
